# Trachoma Prevalence After Discontinuation of Mass Azithromycin Distribution

**DOI:** 10.1093/infdis/jiz691

**Published:** 2020-02-13

**Authors:** William Godwin, Joaquin M Prada, Paul Emerson, P J Hooper, Ana Bakhtiari, Michael Deiner, Travis C Porco, Hamidah Mahmud, Emma Landskroner, T Déirdre Hollingsworth, Graham F Medley, Amy Pinsent, Robin Bailey, Thomas M Lietman, Catherine E Oldenburg

**Affiliations:** 1 Francis I Proctor Foundation, University of California, San Francisco, California, USA; 2 Faculty of Health and Medical Sciences, University of Surrey, Guildford, United Kingdom; 3 International Trachoma Initiative, The Task Force for Global Health, Decatur, Georgia, USA; 4 Department of Ophthalmology, University of California, San Francisco, California, USA; 5 Department of Epidemiology & Biostatistics, University of California, San Francisco, California, USA; 6 Big Data Institute, Li Ka Shing Centre for Health Information and Discovery, University of Oxford, Oxford, United Kingdom; 7 Centre for Mathematical Modelling of Infectious Disease, London School of Hygiene and Tropical Medicine, London, United Kingdom; 8 Faculty of Infectious and Tropical Diseases, London School of Hygiene and Tropical Medicine, London, United Kingdom

**Keywords:** Trachoma, mass drug administration, azithromycin, trachomatous inflammation, follicular

## Abstract

**Background:**

As the World Health Organization seeks to eliminate trachoma by 2020, countries are beginning to control the transmission of trachomatous inflammation–follicular (TF) and discontinue mass drug administration (MDA) with oral azithromycin. We evaluated the effect of MDA discontinuation on TF_1–9_ prevalence at the district level.

**Methods:**

We extracted from the available data districts with an impact survey at the end of their program cycle that initiated discontinuation of MDA (TF_1–9_ prevalence <5%), followed by a surveillance survey conducted to determine whether TF_1–9_ prevalence remained below the 5% threshold, warranting discontinuation of MDA. Two independent analyses were performed, 1 regression based and 1 simulation based, that assessed the change in TF_1–9_ from the impact survey to the surveillance survey.

**Results:**

Of the 220 districts included, TF_1–9_ prevalence increased to >5% from impact to surveillance survey in 9% of districts. Regression analysis indicated that impact survey TF_1–9_ prevalence was a significant predictor of surveillance survey TF_1–9_ prevalence. The proportion of simulations with >5% TF_1–9_ prevalence in the surveillance survey was 2%, assuming the survey was conducted 4 years after MDA.

**Conclusion:**

An increase in TF_1–9_ prevalence may represent disease resurgence but could also be due to measurement error. Improved diagnostic tests are crucial to elimination of TF_1–9_ as a public health problem.

The World Health Organization (WHO) is aiming to achieve the elimination of trachoma as a public health problem by 2020. This goal includes reducing the prevalence of trachomatous inflammation–follicular (TF) in children aged 1–9 years (TF_1–9_) to <5% for ≥2 years in the absence of continued mass drug administration (MDA) in all trachoma-endemic districts (populations, 100 000–250 000 individuals) [1]. MDA with oral azithromycin has been shown to reduce transmission of the ocular strains of chlamydia that cause trachoma [2–4].

To help achieve the elimination of trachoma as a public health problem, the WHO recommends the SAFE strategy (surgery to correct the blinding stage of the disease, antibiotics to clear infection, facial cleanliness, and environmental improvements to reduce transmission), including annual MDA with ≥80% community coverage for communities with baseline TF_1–9_ prevalence >5% for 1–7 years, based on the baseline prevalence within a district [1, 5]. An impact survey is then conducted to determine whether MDA can be stopped. Additional years of SAFE implementation are deemed necessary if this impact survey shows TF_1–9_ prevalence >5%. If TF_1–9_ prevalence is <5%, treatment is discontinued. Two years later, a surveillance survey is conducted to ensure that the TF_1–9_ prevalence has remained <5% (henceforth, “TF_1–9_ control”). Once all formerly endemic districts in a country have remained below the treatment threshold for ≥2 years, the country can prepare a dossier for submission to WHO requesting validation of elimination [6].

Globally, the trachoma program has yielded substantial successes. Since elimination efforts were accelerated approximately 15 years ago, 9 countries have been validated by WHO as having achieved elimination. However, the occurrence of resurgence may undermine the long-term success of trachoma elimination programs, either once TF_1–9_ control has been achieved or if infection and disease returns after several rounds of MDA. To date, in the countries that have been validated by WHO, no resurgence has been reported at the district level; however, understanding factors that may drive resurgence and the epidemiological settings that may facilitate it is of paramount importance for the ongoing success of trachoma elimination.

Here, we perform 2 independent analyses, 1 statistical and 1 mathematical (simulation), to understand the factors that may drive and affect the chance of resurgence, and also the probability under different epidemiological conditions in which resurgence may occur. In the first analysis, we use data provided by the International Trachoma Initiative (ITI) that detail multiple measures of trachoma prevalence and number of treatment rounds through MDA, at the evaluation unit level (henceforth, district level). With these data we investigate how TF_1–9_ prevalence varies in communities after discontinuation of MDA. In our second analysis, we evaluate the probability of stochastic fadeout occurring once TF_1–9_ control has been achieved in the absence of additional rounds of MDA to determine whether measuring prevalence 2 years after stopping MDA is sufficient to that ensure resurgence does not occur.

## METHODS

We used district-level trachoma prevalence and MDA data from the GET2020 Database. Data sources include reports, published articles, and direct contact with nongovernmental organization and national control programs. We included only data from surveys measuring TF_1–9_ prevalence, because children aged 1–9 years are the population on which MDA decisions are based. For a given location-year combination, the database contained, where available, TF_1–9_ prevalence, metadata regarding how TF_1–9_ was surveyed, and total doses of azithromycin distributed. TF_1–9_ prevalence was assessed through population-based cluster random sampling.

Prevalence and treatment data were linked by district-year combination to produce a comprehensive data set of each district’s TF_1–9_ prevalence and treatment status. First, we identified districts with a history of MDA treatment. For each of those districts, all surveys after MDA were identified. Finally, we restricted the data set to include only districts that reported ≥2 surveys after treatment, with no reported rounds of treatment between the surveys. All treatment observations that occurred after the surveillance survey were excluded. In districts with >2 surveys after MDA discontinuation, we included only the 2 most recent surveys. To assess baseline pre-MDA TF_1–9_ prevalence, we identified the initial survey conducted for each district. The final analytic data set thus consisted of 3 surveys for each included district: baseline, impact (impact survey that returned a prevalence estimate for TF_1–9_ of <5% and initiated discontinuation of MDA), and surveillance (surveillance survey conducted after ≥2 years since MDA stopped). The prevalences of TF_1–9_ at the country level and region level (administrative level 2) were calculated by averaging the surveys across all available survey years within the database.

Owing to the long tail to the right of the observed distribution of TF_1–9_ prevalence estimates, all TF_1–9_ data were modeled using square-root transformation. We conducted 2 linear regression models. The first model predicted TF_1–9_ prevalence at the surveillance survey, using TF_1–9_ prevalence at the impact survey. The second model predicted TF_1–9_ prevalence at the surveillance survey, using TF_1–9_ prevalence at the impact survey and the mean TF_1–9_ prevalence of all known trachoma-endemic districts in the country. We used the ordinary least-squares method of estimation and permutation tests to estimate *P* values. All data management and analyses were conducted using R software (version 3.5), and figures were produced using the ggplot2 package [7, 8].

To assess the probability of infection and disease resurging in the community after elimination as a public health problem, we developed a stochastic version of the deterministic transmission model developed by Pinsent et al [9]. Briefly, it is an age-structured, stochastic state-based transmission model reproducing the infection stages of trachoma (susceptible, exposed, diseased and infectious, and diseased but no longer infectious). Individuals in either of the 2 diseased categories were classified as TF_1–9_ positive.

We performed 10 000 stochastic repeats of the model to endemic equilibrium and reproduced the prevalence distribution observed in the ITI data set before intervention. For each simulation, we ran a number of rounds of annual MDA (1–6 rounds), with 80% coverage achieved and a 85% efficacy of the intervention (complete clearance of the infection), and we selected the simulations that achieved TF_1–9_ control (equivalent to the impact survey in the ITI database) after the final round of MDA. We then examined the change in disease prevalence in a population of 10 000 individuals over the next 10 years (equivalent to the surveillance survey). In these simulations, the disease prevalence estimated is the true prevalence in the population of children aged 1–9 years (about 25%–30% of the total population), with no measurement error.

## RESULTS

Of the 1401 districts surveyed and within the GET2020 Database, 220 districts met our inclusion criteria and were included in the analysis. Of the 440 surveys, 337 were labeled as “cluster random sample” and 102 were labeled as “prevalence,” with 1 survey unlabeled. Sixteen countries were represented, and all were located in sub-Saharan Africa, with the exception of Nepal. Baseline surveys across districts were performed from 1996 to 2013 and had a median TF_1–9_ prevalence (interquartile range [IQR]) of 24.3% (14.9%–32.2%) (Table 1). The impact surveys that resulted in the discontinuation of MDA were conducted from 2009 to 2017, with 54.1% (119 of 220) occurring after 2014. The surveillance surveys occurred from 2014 to 2018. The median number of MDA rounds was 3 (IQR, 3–4). The median interval between most recent MDA and the impact survey was 1 year, and the median interval between impact and surveillance surveys was 2 years. Eleven of the 220 included districts had ≥3 surveys after MDA discontinuation.

**Table 1.** Survey Periods and Trachomatous Inflammation–Follicular Prevalence for Surveys Included in the Analysis

	Median (IQR)
Survey period	
Baseline	2007 (2003–2011)
Impact survey	2015 (2012–2016)
Surveillance survey	2017 (2017–2018)
Interval between surveys, y	2 (2–4)
TF_1–9_ prevalence, %	
Baseline	24.3 (14.9–32.2)
Impact survey	1.7 (0.9–2.8)
Surveillance survey	1.13 (0.5–2.0)
Change between surveys	1.0 (0.4–2.0)

Abbreviations: IQR, interquartile range; TF, trachomatous inflammation–follicular.

The median TF_1–9_ prevalence (IQR) at the country level was 10.6% (9.2%–13.5%) (Table 2). Across districts, the median TF_1–9_ prevalences were 1.7% (IQR, 0.9%–2.8%) and 1.1% (0.5%–2.0%) for the impact and surveillance surveys, respectively. The Pearson correlation coefficient on square-root transformed TF_1–9_ prevalence was 0.36 between the impact and surveillance surveys. All 220 districts reported TF_1–9_ control at the impact survey, and 91% (201 of 220) reported TF_1–9_ control at the surveillance survey. The TF_1–9_ prevalence increased above the control threshold in 9% of districts (19 of 220) from the impact to the surveillance survey. Among the 19 districts where TF_1–9_ prevalence climbed above the control threshold, prevalence remained <10% in 9 districts and rose to >10% in 10, with a median increase of 6.3% (IQR, 3.4%–9.3%). Comparing the 19 districts with the districts that remained at control threshold, the mean numbers of MDA rounds were 3.6 (range, 2–9) and 3.5 (1–9), respectively, and the average intervals between MDA and the impact survey were 1.1 (1–2) and 1.5 (1–7) years, respectively.

**Table 2.** Characteristics of Districts Included in the Analysis

Characteristic	Median (IQR)
Survey period	2016 (2015–2017)
MDA rounds, no.^a^	3 (3–4)
Interval between MDA and surveillance survey, y	4 (3–7)
Country prevalence of TF_1–9_, %	10.6 (9.2–13.5)

Abbreviation: IQR, interquartile range; MDA, mass drug administration; TF, trachomatous inflammation–follicular.

^a^Number of annual azithromycin MDA rounds preceding the impact survey.

**Figure 1.**
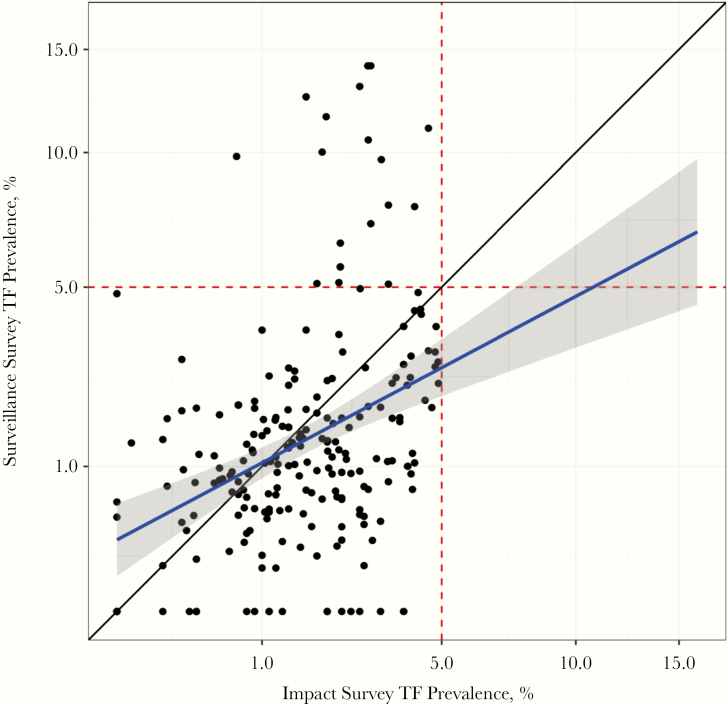
Prevalence of trachomatous inflammation–follicular in children aged 1–9 years (TF_1–9_) at the impact and surveillance surveys, with fitted regression line shown in blue. Red dashed lines denote TF_1–9_ control threshold; black line, 45º line. The axes are square-root transformed.

**Figure 2.**
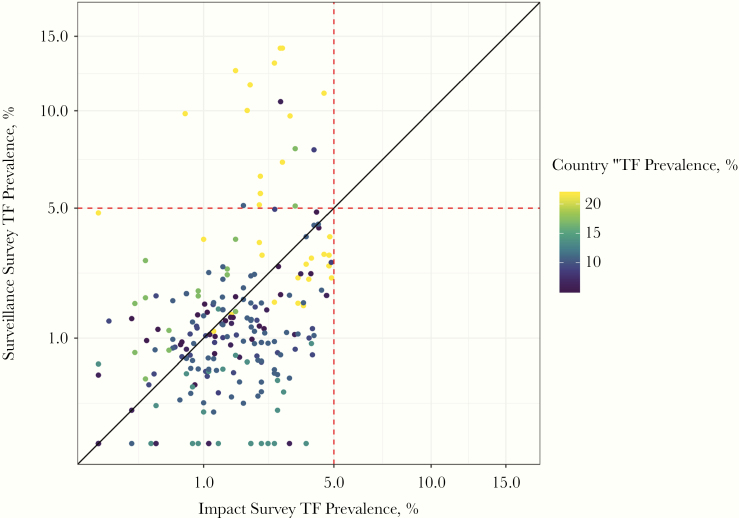
Prevalence of trachomatous inflammation–follicular in children aged 1–9 years (TF_1–9_) in impact and surveillance surveys (square-root scale). Points are colored according to country TF_1–9_ prevalence. Red dashed lines denote TF_1–9_ control threshold; black line, 45º line. The axes are square-root transformed.

From the first linear regression model (Figure 1), we found that higher TF_1–9_ prevalence at the impact survey was associated with significantly higher TF_1–9_ prevalence at the surveillance survey (Table 3). In addition, as country TF_1–9_ prevalence increased, so did TF_1–9_ prevalence at the surveillance survey. Figure 2 illustrates the relationship between country-level TF_1–9_ prevalence and TF_1–9_ prevalences from impact and surveillance surveys. In a sensitivity analysis, we replaced the country-level TF_1–9_ indicator with region-level (administrative level 1) TF_1–9_ prevalence in the linear regression and found quantitatively similar results (Supplementary Figure 1).

**Table 3.** Linear Regression Coefficients Produced from Modeling Square-Root Transformed Values

Model and Term^a^	β Coefficient (95% CI)
Model 1	
Intercept	.49 (.24–.74)
Impact survey TF_1–9_ prevalence	.53 (.35–.71)
Model 2	
Intercept	−.07 (−.34 to .20)
Impact survey TF_1–9_ prevalence	.41 (.23–.59)
Country TF_1–9_ prevalence	.06 (.04–.08)

Abbreviations: CI, confidence interval; TF, trachomatous inflammation–follicular.

^a^Model 1 is a simple linear regression model with the surveillance survey TF_1–9_ prevalence regressed on the impact survey TF_1–9_ prevalence. Model 2 is a multiple linear regression model with the surveillance survey TF_1–9_ prevalence regressed on the impact survey and country-level TF_1–9_ prevalences.

**Figure 3.**
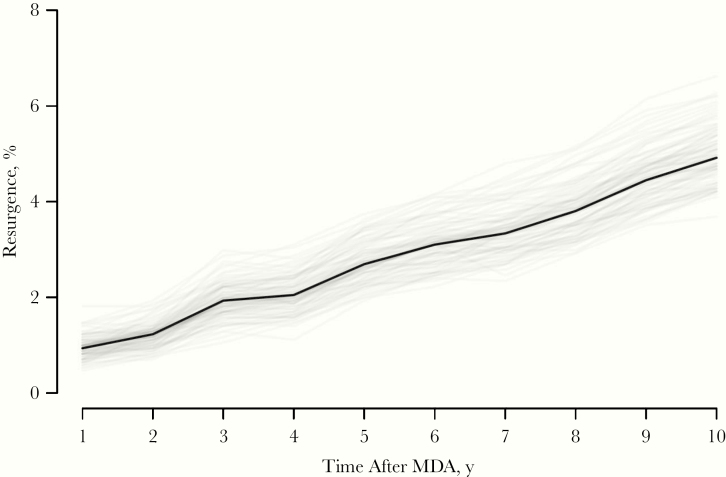
Rate of true resurgence after stopping of mass drug administration (MDA) and achievement of trachomatous inflammation–follicular (TF) control and prevalence >5% for TF_1–9_ (*black line*). Over time, more simulations show regurgence, until a new equilibrium is reached (not reached 10 years after MDA). Simulations that required more rounds of MDA to reach TF_1–9_ control are more likely to resurge. Gray lines represent 100 bootstrapped values obtained from sampling the runs with replacement.

In the simulations of the stochastic model, reproducing the conditions of the ITI database, the observed proportion of simulations in which prevalence increased above TF_1–9_ control was 5% (Figure 3). This value was achieved when the time between impact and surveillance survey was 10 years (ie, the maximum interval that we considered). In the ITI database, the median interval between the surveys is 4 years, which yields a 2.1% true resurgence observed.

## DISCUSSION

In 9% of formerly trachoma-endemic districts with ≥2 surveys after discontinuation of azithromycin MDA, TF_1–9_ prevalence increased above the threshold for TF_1–9_ control. There are 2 likely drivers for apparent resurgence in these 19 districts. The first possibility is misclassification of TF_1–9_ through regression to the mean or stochasticity in the measurement. Owing to the array of survey methods used, lack of population representativeness, and TF_1–9_ grader drift (inherent subjectivity of TF_1-9_ diagnosis), TF_1–9_ surveys are often subject to large amounts of noise, particularly in areas of low prevalence [3, 10]. This may be the mechanism underlying the 9 districts in which TF_1–9_ prevalence increased above control but remained <10%. The second potential driver of the districts that increased to >10% TF_1–9_ prevalence is true resurgence of TF_1–9_. Especially in countries with higher background prevalence of infection, resurgence could occur, for example, via migration from affected communities to communities that had previously achieved control. More accurate methods to distinguish between misclassification and true resurgence, such as masking graders to geographic area or conducting algorithm-based automated assessment of eyelid photographs [11], will be necessary in order to identify districts requiring additional intervention.

Based on the correlation between the impact and surveillance surveys, 14% of the variation in TF_1–9_ prevalence in the surveillance (second) survey can be explained using TF_1–9_ prevalence in the impact (first) survey. The remaining 86% of variation in impact survey TF_1–9_ prevalence may be explained by a number of factors, including misclassification, mentioned above, true fluctuations in TF_1–9_, and changing hygiene practices that affect transmission dynamics. Variation in MDA coverage in districts may explain some differences in TF_1–9_ prevalence, but we did not have access to coverage data and could not evaluate its effect on TF_1–9_ prevalence. In the simulation-based model, MDA coverage was assumed to be 80% with 85% efficacy.

The results from the simulation-based model, reproduced using the conditions of the ITI data set, suggest that true resurgence is lower than reported, with only 5% of the simulations going above TF_1–9_ control 10 years after discontinuation of yearly MDA. Moreover, in these simulations from the simulation-based model, we did not include improvement of sanitation, hygiene, and other factors that would decrease transmission over time.

We observed 9% of districts increase above TF_1–9_ control within the ITI data set, whereas results of the simulation-based model indicate that only 2% of districts would resurge above TF_1–9_ control given similar conditions. Both indicate potential resurgence and the importance of monitoring this possibility in the future. The model further illustrates how stochastic measurements can lead to misclassification of TF_1–9_. Future simulation-based models can investigate the influence of measurement error, sampling noise, and hygiene practices on the probability of resurgence.

We hypothesize that greater differences in TF_1–9_ prevalence between surveys is more likely to indicate resurgence, and small changes are likely due to noise; however, development of complementary methods to distinguish between misclassification and resurgence would be required to test this hypothesis. Currently, the only available tool to measure ocular *Chlamydia trachomatis* infection is conjunctival swabbing and polymerase chain reaction to identify organisms. Although accurate and the reference standard measure for identifying infection, polymerase chain reaction is not logistically feasible outside of clinical trials, owing to the high cost of processing samples and the requirement of a cold chain to transport samples. An alternative is the use of serology to measure antibodies to *C. trachomatis* [12, 13]*. C. trachomatis* antigens Pgp3 and CT694 have been shown to correlate with both infection and disease status [13, 14]. Finally, to address potential biases in measuring TF_1–9_ using subjective graders, there is some promise in using conjunctival photography to measure active trachoma [15]. Photographs may reduce some bias introduced by field graders, allowing grades to be assigned in a standardized fashion masked to geographic area and treatment history.

Our analysis had several limitations. The clinical sign of TF_1–9_ does not reliably indicate *C. trachomatis* infection, because TF_1–9_ often persists for weeks or months after the infection has cleared [16–18]. Grading of TF_1–9_ is subjective and lacks reliability, with intraclass correlation often reported to be <70% [15]. Owing to the nature of the evaluation units defined by MDA programs, the spatial criteria that defined a district varied slightly, with a few subdistricts included in the analysis. In addition, we analyzed MDA treatment as a binary indicator, whereas coverage is likely variable across districts and may help predict TF_1–9_ prevalence. Most critically, our study could not distinguish between misclassification and true resurgence of TF_1–9_ in cases where TF_1–9_ prevalence increased.

Global health campaigns of mass antibiotic distribution have been largely successful in reducing TF_1–9_ prevalence to control levels in previously endemic areas. As more districts control and eliminate TF_1–9_, evaluation and monitoring for the resurgence of TF_1–9_ is important in order to maintain elimination. The resurgence of TF_1–9_ reported from survey data could indicate the return of ocular *C. trachomatis* transmission and could lead to additional rounds of MDA but may also simply represent noise and substantial stochastic variation in the data. In the current report, we presented evidence that a small minority of districts may be experiencing an increase in TF_1–9_ prevalence, which could lead to resurgence of transmission. Improved diagnostic tests for TF_1–9_ are necessary to determine whether this result was due to misclassification or true resurgence.

## Supplementary Data

Supplementary materials are available at *The Journal of Infectious Diseases* online. Consisting of data provided by the authors to benefit the reader, the posted materials are not copyedited and are the sole responsibility of the authors, so questions or comments should be addressed to the corresponding author.

jiz691_suppl_Supplemental_Table

## Notes

***Financial support.*** This work was supported by the Bill & Melinda Gates Foundation (https://www.gatesfoundation.org) in partnership with the Task Force for Global Health (https://www.taskforce.org) through the NTD Modeling Consortium (grant OPP1184344 to T. M. L.); by the National Eye Institute (https://nei.nih.gov) (grant EY025350 to T. M. L., grant L40-EY028421 to C. E. O., and core grant EY002162); and by Research to Prevent Blindness (https://www.rpbusa.org; unrestricted grant and career development award to C. E. O.).

***Supplement sponsorship.*** The authors acknowledge funding of the NTD Modelling Consortium by the Bill and Melinda Gates Foundation (OPP1184344).

***Potential conflicts of interest.*** All authors: No reported conflicts. All authors have submitted the ICMJE Form for Disclosure of Potential Conflicts of Interest. Conflicts that the editors consider relevant to the content of the manuscript have been disclosed.

## References

[1] World Health Organization. Report of the third global scientific meeting on trachoma 2010. https://www.who.int/trachoma/resources/who_pbd_2.10/en/. Accessed 2 October 2019.

[2] Schachter J, West SK, Mabey D, et al Azithromycin in control of trachoma. Lancet 1999; 354:630–5. 10466664 10.1016/S0140-6736(98)12387-5

[3] Solomon AW, Peeling RW, Foster A, Mabey DCW Diagnosis and assessment of trachoma. Clin Microbiol Rev 2004; 17:982–1011. 15489358 10.1128/CMR.17.4.982-1011.2004 PMC523557

[4] Chidambaram JD, Alemayehu W, Melese M, et al. Effect of a single mass antibiotic distribution on the prevalence of infectious trachoma. JAMA 2006; 295:1142–6. 16522834 10.1001/jama.295.10.1142

[5] Thylefors B, Dawson CR, Jones BR, West SK, Taylor HR A simple system for the assessment of trachoma and its complications. Bull World Health Organ 1987; 65:477–83. 3500800 PMC2491032

[6] World Health Organization. Validation of elimination of trachoma as a public health problem 2016. https://www.who.int/neglected_diseases/resources/who_htm_ntd_2016.8/en/. Accessed 2 October 2019.

[7] R Core Team, R Foundation for Statistical Computing. R: a language and environment for statistical computing. Vienna, Austria: 2018 https://www.r-project.org/. Accessed 17 April 2019.

[8] Wickham H. ggplot2: Elegant graphics for data analysis. New York, NY: Springer-Verlag, 2009 http://ggplot2.org. Accessed 17 April 2019.

[9] Pinsent A, Liu F, Deiner M, et al. Probabilistic forecasts of trachoma transmission at the district level: a statistical model comparison. Epidemics 2017; 18:48–55. 28279456 10.1016/j.epidem.2017.01.007 PMC5340843

[10] Thein J, Zhao P, Liu H, et al. Does clinical diagnosis indicate ocular chlamydial infection in areas with a low prevalence of trachoma? Ophthalmic Epidemiol 2002; 9:263–9. 12187424 10.1076/opep.9.4.263.1508

[11] Kim MC, Okada K, Ryner AM, et al. Sensitivity and specificity of computer vision classification of eyelid photographs for programmatic trachoma assessment. PLoS One 2019; 14:e0210463. 30742639 10.1371/journal.pone.0210463 PMC6370195

[12] Martin DL, Wiegand R, Goodhew B, et al. Serological measures of trachoma transmission intensity. Sci Rep 2015; 5:18532. 26687891 10.1038/srep18532 PMC4685243

[13] Goodhew EB, Priest JW, Moss DM, et al. CT694 and pgp3 as serological tools for monitoring trachoma programs. PLoS Negl Trop Dis 2012; 6:e1873. 23133684 10.1371/journal.pntd.0001873 PMC3486877

[14] Kim JS, Oldenburg CE, Cooley G, et al. Community-level chlamydial serology for assessing trachoma elimination in trachoma-endemic Niger. PLoS Negl Trop Dis 2019; 13:e0007127. 30689671 10.1371/journal.pntd.0007127 PMC6366708

[15] Gebresillasie S, Tadesse Z, Shiferaw A, et al. Inter-rater agreement between trachoma graders: comparison of grades given in field conditions versus grades from photographic review. Ophthalmic Epidemiol 2015; 22:162–9. 26158573 10.3109/09286586.2015.1035792 PMC4802863

[16] See CW, Alemayehu W, Melese M, et al How reliable are tests for trachoma? a latent class approach. Investig Ophthalmol Vis Sci 2011; 52:6133–7. 21685340 10.1167/iovs.11-7419 PMC3176003

[17] Koukounari A, Moustaki I, Grassly NC, et al. Using a nonparametric multilevel latent Markov model to evaluate diagnostics for trachoma. Am J Epidemiol 2013; 177:913–22. 23548755 10.1093/aje/kws345 PMC3639724

[18] Bailey RL, Arullendran P, Whittle HC, Mabey DC Randomised controlled trial of single-dose azithromycin in treatment of trachoma. Lancet 1993; 342:453–6. 8102427 10.1016/0140-6736(93)91591-9

