# Erratum to: Trachoma Prevalence After Discontinuation of Mass Azithromycin Distribution

**DOI:** 10.1093/infdis/jiaa082

**Published:** 2020-03-26

**Authors:** William Godwin, Joaquin M Prada, Paul Emerson, P J Hooper, Ana Bakhtiari, Michael Deiner, Travis C Porco, Hamidah Mahmud, Emma Landskroner, T Deirdre Hollingsworth, Graham F Medley, Amy Pinsent, Robin Bailey, Thomas M Lietman, Catherine E Oldenburg

**Affiliations:** 1 Francis I Proctor Foundation, University of California, San Francisco, California, USA; 2 Faculty of Health and Medical Sciences, University of Surrey, Guildford, United Kingdom; 3 International Trachoma Initiative, The Task Force for Global Health, Decatur, Georgia, USA; 4 Department of Ophthalmology, University of California, San Francisco, California, USA; 5 Department of Epidemiology & Biostatistics, University of California, San Francisco, California, USA; 6 Big Data Institute, Li Ka Shing Centre for Health Information and Discovery, University of Oxford, Oxford, United Kingdom; 7 Centre for Mathematical Modelling of Infectious Disease, London School of Hygiene and Tropical Medicine, London, United Kingdom; 8 Faculty of Infectious and Tropical Diseases, London School of Hygiene and Tropical Medicine, London, United Kingdom

In “Trachoma Prevalence After Discontinuation of Mass Azithromycin Distribution [J Infect Dis. 2020 Feb 13, jiz691, https://doi.org/10.1093/infdis/jiz691]” by Godwin et al., the first sentence of the Results section includes a reference to “ITI database” that is incorrect and should read as “GET2020 Database”. In addition, the authors note that major contributors to the GET2020 database include numerous Ministries of Health worldwide as well as the Global Trachoma Mapping Project (Solomon AW, Pavluck AL, Courtright P, et al. The Global Trachoma Mapping Project: Methodology of a 34-Country Population-Based Study. Ophthalmic Epidemiol 2015; 22(3):214–25). The authors regret the error.

