# Supplementary material for: Trachoma Prevalence After Discontinuation of Mass Azithromycin Distribution
Source: J Infect Dis. 2020 Feb 13;221(Suppl 5):S519–24. doi: 10.1093/infdis/jiz691 (PMC7289551; doi:10.1093/infdis/jiz691)
Supplement: jiz691_suppl_Supplemental_Table [file jiz691_suppl_supplemental_table.docx]

**Table S1.** Model results after replacing Country TF with Region TF. Regression coefficients produced from modeling square-foot transformed values. The model was a multiple linear regression model with surveillance survey TF regressed on impact survey TF and region-level TF.

| **Term** | **β-coefficient** | **95% CI** |
| --- | --- | --- |
| Intercept | 0.04 | -0.23 - 0.31 |
| Impact survey TF | 0.37 | 0.19 - 0.55 |
| Region TF | 0.06 | 0.04 - 0.08 |
